# Graphene-Based Perfect Absorption Structures in the Visible to Terahertz Band and Their Optoelectronics Applications

**DOI:** 10.3390/nano8121033

**Published:** 2018-12-12

**Authors:** Chucai Guo, Jianfa Zhang, Wei Xu, Ken Liu, Xiaodong Yuan, Shiqiao Qin, Zhihong Zhu

**Affiliations:** College of Advanced Interdisciplinary Studies, National University of Defense Technology, Changsha 410073, China; jfzhang85@nudt.edu.cn (J.Z.); weixu08a@163.com (W.X.); liukener@163.com (K.L.); xyuan@nudt.edu.cn (X.Y.); sqqin8@nudt.edu.cn (S.Q.)

**Keywords:** graphene, perfect absorption, optoelectronic devices

## Abstract

Graphene has unique properties which make it an ideal material for photonic and optoelectronic devices. However, the low light absorption in monolayer graphene seriously limits its practical applications. In order to greatly enhance the light absorption of graphene, many graphene-based structures have been developed to achieve perfect absorption of incident waves. In this review, we discuss and analyze various types of graphene-based perfect absorption structures in the visible to terahertz band. In particular, we review recent advances and optoelectronic applications of such structures. Indeed, the graphene-based perfect absorption structures offer the promise of solving the key problem which limits the applications of graphene in practical optoelectronic devices.

## 1. Introduction

Graphene is a novel two-dimensional (2D) material, which has been studied widely due to its unique properties. Graphene has the ultrabroad absorption spectrum [1], the ultrafast response speed towards light and the tunable conductivity [2,3], which make it an ideal material for photonic and optoelectronic devices, such as ultrafast graphene photodetectors [4,5,6,7] and optical modulators [8,9,10,11,12,13,14]. However, the light absorption efficiency of monolayer graphene is weak [15], which seriously limits its applications in practical optoelectronic devices.

In order to profoundly enhance the light absorption of graphene, many graphene-based perfect absorption structures have been developed. In the microwave, the absorption of graphene can be easily tuned by controlling the carrier density of graphene, and perfect absorption of graphene has been experimentally realized by combing a graphene capacitor with a metallic surface [16] or by employing coherent illumination [17]. In the mid-infrared (IR) to terahertz (THz) band, graphene supports plasmon resonances, which can greatly enhance the near-field of graphene. Thus, many perfect absorption structures based on graphene plasmons have been proposed, such as narrowband perfect absorption structure in the mid-IR [18], dual-band perfect absorption structures in the THz [19,20], multi-band perfect absorption structures in the mid-IR [21,22], broadband perfect absorption structures in the THz [23,24], and coherent perfect absorption structures in the mid-IR and THz [25,26]. Meanwhile, without exciting the graphene plasmons, some graphene-based perfect absorption structures were also demonstrated by using the impedance matching concept [27] or special designs [28,29,30] in the THz band, and by using the big incident angle in the mid-IR band [31]. In the visible and near-IR band, graphene can hardly support plasmons due to the limits of doping or gating level, and the absorption of graphene was normally enhanced by coupling graphene with metallic or dielectric resonant structures. In this band region, graphene-based perfect absorption has been demonstrated by utilizing localized surface plasmon resonances of metals [32,33,34,35,36,37,38,39,40,41,42,43], Fabry–Pérot cavity resonances [44,45], photonic crystal cavity mode [46], guided mode resonances [47,48,49,50,51,52,53,54,55,56,57,58,59,60,61,62,63,64,65,66], or by using prism coupling [67,68], aperiodic multilayer microstructures [69] and coherent illumination [70,71].

In the application aspect, graphene-based perfect absorption structures have great potential in optoelectronic devices. Graphene-based perfect absorption structures can effectively enhance the responsivity of graphene photodetectors [44,45] and improve the modulation depth of graphene modulators [72,73]. Meanwhile, electrically tunable optical filters [74,75] and polarizers [76,77], and refractive index sensors [56] have also been proposed by using such structures.

In this paper, we will give a comprehensive review of graphene-based perfect absorption structures in the visible to THz band. Firstly, we will introduce the theory of perfect absorption. Secondly, we discuss and analyze various types of graphene-based perfect absorption structures in the mid-IR to THz band and in the visible to near-IR band, respectively. The recent progress of those structures will also be introduced. Still next, we review the optoelectronic applications of graphene-based perfect absorption structures. Finally, we will highlight the perspectives and challenges of graphene-based perfect absorption structures.

## 2. Perfect Absorption Theory

Perfect absorption phenomenon has been studied for many years, here we will introduce the perfect absorption theory by using two simple structures. The first structure is a single mode resonator with one-side input wave, and the resonator can perfectly absorb the input wave when the critical coupling condition is satisfied. The second structure consists of an ultra-thin absorbing sheet (e.g., graphene) and a dielectric spacer on top of a thick metal layer, and perfect absorption can be realized by matching the impedance of the structure to that of vacuum.

### 2.1. Critical Coupling Concept

It’s well known that the resonant effect can effectively enhance the optical absorption of materials due to the local field enhancement, and resonant structures have been widely utilized to realize perfect absorption of an incident wave. In the following, we will discuss the perfect absorption conditions for a single mode resonator.

**Figure 1:**
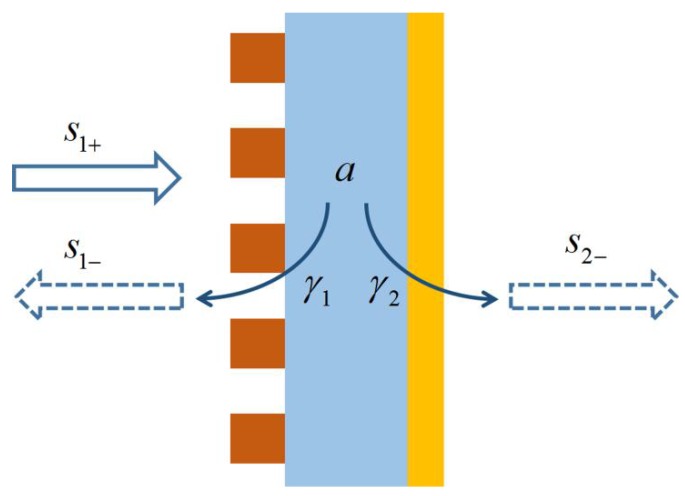
Single mode resonator with one-side input wave.

A normal single mode resonator with one-side input wave, as shown in Figure 1, can be described by the following equations according to the coupled mode theory [78]:

$$\dot{a}=-j\omega_{0}a-a\gamma_{1}-a\gamma_{2}-a\gamma_{a}+\sqrt{2\gamma_{1}}s_{1+}$$

(1) $$s_{1-}=-s_{1+}+\sqrt{2\gamma_{1}}a$$

$$s_{2-}=\sqrt{2\gamma_{2}}a$$

where a denotes the mode amplitude of the resonator. $s_{1+}$ denotes the input field amplitude, $s_{1-}$ and $s_{2-}$ denote the reflection and transmission field amplitude, respectively. $\gamma_{1}$ and $\gamma_{2}$ denote the mode leakage rate to the front side and back side of the resonator, respectively. $\gamma_{a}$ denotes the absorption rate of the resonator, $\omega_{0}$ is the resonance frequency. The reflection and transmission coefficients of the system are $s_{1-}$/$s_{1+}$ and $s_{2-}$/$s_{1+}$, respectively. By solving the Equation (1), we can get the absorption of the system:

(2) $$A_{b}=1-{\left|\frac{s_{1-}}{s_{1+}}\right|}^{2}-{\left|\frac{s_{2-}}{s_{1+}}\right|}^{2}=\frac{4\gamma_{a}\gamma_{1}}{{\left(\gamma_{1}+\gamma_{2}+\gamma_{a}\right)}^{2}+{\left(\omega -\omega_{0}\right)}^{2}}$$

In order to get perfect absorption ($A_{b}$ = 1) at resonant frequency ($\omega =\omega_{0}$), the transmission of the resonator should be blocked ($\gamma_{2}=0$), and the mode leakage rate $\gamma_{1}$ must be equal to the absorption rate $\gamma_{a}$, which is called the critical coupling condition.

When a perfect absorption structure is designed by integrating graphene with a resonator illuminated from one side, a back mirror is needed to completely block the transmission. In order to satisfy the critical coupling condition ($\gamma_{1}$ = $\gamma_{a}$), the quality factor (*Q* factor) of the resonator should be controlled carefully, since it determines the mode leakage rate $\gamma_{1}$. The absorption rate of graphene is determined by the graphene absorption coefficient (a constant, without considering the nonlinear effects) and the field intensity in the graphene layer. Therefore, both the field distribution of the resonant mode and the position of graphene in the resonator will affect the absorption rate $\gamma_{a}$. If we want to design a narrow band perfect absorption structure, a high-*Q* resonator is needed and the graphene should be away from the place with high field intensity. Conversely, we should concentrate the mode field near the graphene and reduce the *Q* factor of the resonator.

### 2.2. Impedance Matching Concept

Critical coupling concept depends on the coupling of the incident wave with a resonant mode, however, perfect absorption can be also realized without a resonant mode. In the following, we will introduce the impedance matching concept to achieve perfect absorption, whereas a resonant mode is unnecessary.

**Figure 2:**
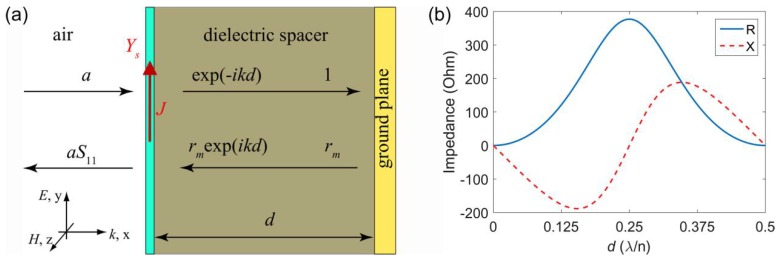
(**a**) Schematic illustration of the absorption structure which comprises a ultra-thin sheet, dielectric spacer and metallic ground plane. The amplitude of *E* field of forward going wave at the ground plane is chosen to be 1 while the amplitude of incident *E* field is denoted as *a*. (**b**) The resistance (*R*) and reactance (*X*) of the impedance for PIMS as a function of the dielectric spacer thickness. Reproduced from reference [79], with permission from Optical Society of America, 2011.

When a ultra-thin absorbing sheet is placed in front of a thick metal layer (denoted as ground plane) with a distance d, as shown in Figure 2a, perfect absorption of the structure can be realized by matching the impedance of the structure to that of vacuum, and in this case, the sheet is called a perfectly impedance-matched sheet (PIMS) [79].

According to the boundary conditions for the ultra-thin sheet under normal incidence condition, we can get the following Equation:

$$a+aS_{11}=\exp\left(-ikd\right)+r_{m}\exp\left(ikd\right)$$

(3) $$Y_{0}\left(a-aS_{11}\right)=Y_{1}\left(\exp\left(-ikd\right)-r_{m}\exp\left(ikd\right)\right)+J$$

$$J=Y_{s}\left(a+aS_{11}\right)$$

where a is the amplitude of incident electric field, $S_{11}$ is the reflection coefficient of the structure. $Y_{0}$ is the admittance of vacuum, $Y_{1}=\sqrt{\varepsilon_{1}}Y_{0}$ and $Y_{m}=\sqrt{\varepsilon_{m}}Y_{0}$ are the admittance of dielectric and metal, $\varepsilon_{1}$ and $\varepsilon_{m}$ are permittivities of dielectric and metal. $k=\sqrt{\varepsilon_{m}}k_{0}$ is the wave vector in the dielectric spacer, and $k_{0}$ is the wave vector in vacuum. $r_{m}=\left(Y_{1}-Y_{m}\right)/\left(Y_{1}+Y_{m}\right)$ is the reflection coefficient of the metal layer, and J is the current in the ultra-thin sheet.

For perfect absorption, $S_{11}$ should be zero, thus the admittance of PIMS can be obtained by eliminating a in Equation (3):

(4) $$Y_{S}=Y_{0}-Y_{1}\frac{\exp(-ikd)-r_{m}\exp(ikd)}{\exp(-ikd)+r_{m}\exp(ikd)}$$

The resistance (*R*) and reactance (*X*) of the impedance for PIMS (*Z*_S_ = *R* − i*X*) can be obtained from Equation (4). The values of *R* and *X* as a function of the spacer thickness *d* are plotted in Figure 2b, where the refractive index of the dielectric spacer *n* is chosen as 1.45 (corresponding to refractive index of silica in the IR) and *r*_m_ is chosen as −1(corresponding to reflection coefficient of perfect electric conductor).

Considering graphene as the absorbing sheet in Figure 2a, the impedance of monolayer graphene (*Z*_S_ = 1/σ_2D_(ω), σ_2D_(ω) is the conductivity of graphene) in the visible to near-IR band cannot satisfy Equation (4), since the real part of the graphene conductivity in this band is close to 6.08 × 10^−5^ Ω^−1^ [80,81]. However, the impedance of highly doped graphene [27] and the effective impedance of patterned graphene [82] in the THz can satisfy Equation (4). Therefore, the impedance matching concept can be utilized to design graphene-based perfect absorption structures in the THz.

## 3. Perfect Absorption in the Mid-IR to THz Band

### 3.1. Graphene Plasmons Induced Perfect Absorption

Plasmons are collective density oscillations of free electrons in metals and semiconductors. Doped graphene can support plasmons in the mid- and far-IR to THz band, and graphene plasmons have many advantages over plasmons in metals [83,84,85]. Firstly, free carriers in graphene can be induced by electrostatic gating or doping, so graphene plasmons can be easily tuned by gating or doping. Secondly, plasmons in highly crystalline graphene have lifetimes over hundreds of optical cycles, so the graphene plasmons have low losses compared with plasmons in metals. Thirdly, the plasmon wave vector in graphene can be two orders larger than the wavevector in vacuum, so the graphene plasmons have extreme confinement.

**Figure 3:**
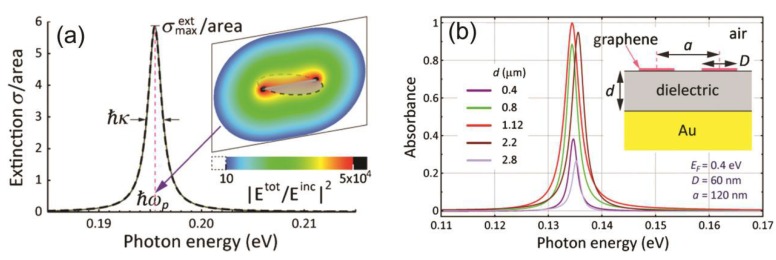
(**a**) Characteristic extinction cross section of a nanodisk (60 nm in diameter and Fermi energy *E*_F_ = 0.4 eV), dominated by a pronounced dipolar plasmon and strong near-field enhancement. Solid curve: Full electrodynamic calculation. Dashed curve: Fit to a Lorentzian polarizability. Inset: Near-field intensity normalized to incident intensity for plane-wave illumination with polarization parallel to the disk. (**b**) Normal-incidence absorbance by graphene disk arrays supported on a dielectric-coated gold surface (see the inset) for various values of the dielectric film thickness. Reproduced from reference [87], with permission from American Physical Society, 2012.

Graphene plasmons can greatly enhance the near-field of graphene, which can be utilized to enhance the absorption [86] or realize perfect absorption of input light. In 2012, Thongrattanasiri et al. numerically demonstrated that 100% light absorption can occur in a layer of doped graphene nanodisks [87]. As shown in Figure 3a, doped graphene nanodisks support a dipolar plasmon which provides strong near-field enhancement. Therefore, graphene absorption can be greatly enhanced. However, the maximum absorption of graphene disks in a symmetric environment is limited to 50%, so asymmetric environments are needed to further increase the graphene absorption. The authors designed an absorption structure by putting the graphene nanodisks on top of a dielectric-coated gold substrate [see the inset in Figure 3b]. The transmission of the structure is totally suppressed by the gold substrate, so perfect absorption can be realized if the reflection is canceled. Simulation results demonstrated that 100% absorption in graphene is achieved by properly choosing the coating-layer thickness d to satisfy the critical coupling condition, as shown in Figure 3b.

Until now, single-band [88,89,90], dual-band [91,92,93] and multi-band [94,95] perfect absorption structures based on graphene plasmons by using patterned or unpatterned [90,96] graphene in the mid-IR to THz band have been demonstrated. The absorption wavelength and absorbance of those structures can be dynamically tuned by the gating level of graphene [97,98,99,100,101,102], which is the key advantage compared with metallic perfect absorption structures.

Nevertheless, the graphene plasmons induced perfect absorption discussed above is normally band-limited or narrowband, which is restricted in some broadband applications. In the following, we will discuss graphene-based broadband perfect absorption structures.

### 3.2. Broadband Perfect Absorption

Broadband graphene-based perfect absorption is highly desirable for some applications, such as imaging and photodetection. In order to achieve broadband perfect absorption in the mid-IR to THz band, various approaches have been investigated. One effective method is using two layers or multi-layers patterned graphene to increase the absorption bandwidth [23,103,104,105,106,107,108,109]. In 2013, A. Andryieuski et al. demonstrated a broadband perfect absorption structure by using graphene fishnet metamaterial which comprises two layers of structured graphene separated with a very thin dielectric. Numerical results show that the absorption bandwidth of the structure with absorbance over 90% is about 1.9 THz at central frequency around 3.1 THz, and the normalized bandwidth (NBW) is about 61.3% [23]. M. Amin et al. proposed an ultra-broadband perfect absorption structure by using three layers of patterned graphene, and the absorption of the designed structure is over 90% in the frequency range of 4.7 THz to 11.6 THz (NBW = 84.7%) [103]. Recently, a perfect absorption structure with absorbance over 90% in the frequency band ranging from 0.55 to 3.12 THz (NBW = 140%) was also designed by using three layers of differently shaped graphene patterns [107]. However, utilizing multi-layers of graphene is not beneficial to the miniaturization of the absorption structures.

Another effective method to achieve broadband absorption is using multi-resonances of patterned monolayer graphene [110,111,112,113,114,115]. In 2014, Z. H. Zhu et al. demonstrated a broadband perfect absorption structure by using single-layered elliptical graphene ribbons. The designed periodic array of doped graphene ribbons with gradient width supports three plasmon resonances, which can effectively extend the absorption bandwidth. Numerical results demonstrated that the bandwidth with absorbance over 90% is as high as 1.3 THz at central frequency of 3 THz [110]. The structure based on graphene ribbons is sensitive to incident polarization, and then polarization-insensitive or polarization-independent broadband absorption structures were designed by using sinusoidally-patterned graphene [111], cross-oval-shaped graphene [112] and graphene concentric double ring [113].

Meanwhile, monolayer-graphene-based broadband perfect absorption structures were demonstrated by using the impedance matching concept [82,116,117]. Also, absorption structures with an NBW of about 100% in the THz were demonstrated by using graphene ribbons [82] and graphene disks [116].

**Figure 4:**
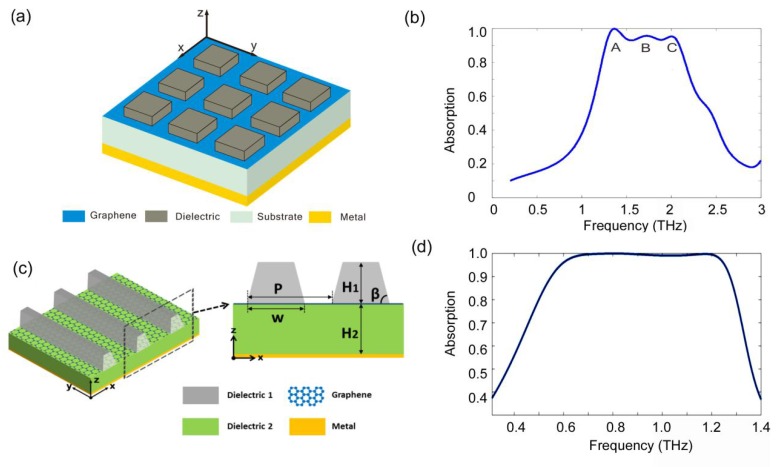
(**a**) Schematic depiction of the absorption structure by coupling unpatterned graphene with dielectric bricks. (**b**) Calculated absorption spectrum of the designed structure shown in (**a**) under normal incidence [118]. (**c**) Schematic illustration of the absorption structure by coupling unpatterned graphene with dielectric wires. (**d**) Calculated absorption spectrum of the designed structure shown in (**c**) for TM polarization under normal incidence [24]. Reproduced from references [24,118], with permission from Optical Society of America, 2018; with permission from Optical Society of America, 2017.

The broadband absorption structures discussed above are based on patterned graphene which will result in some edge effects, thus rendering it difficult to realize perfect absorption in reality. In order to avoid the edge effects, broadband perfect absorption structures based on unpatterned graphene are put forward [24,118,119]. In 2017, F. Gao et al. demonstrated a broadband perfect absorption structure by using single-layered unpatterned graphene [118]. The schematic image of the proposed structure is shown in Figure 4a, which consists of single-layered graphene sandwiched between 2D periodic dielectric bricks and a substrate, and a thick layer of metal is coated on the back side of the substrate. Figure 4b shows the calculated absorption spectrum of a designed structure for transverse-electric (TE) polarization under normal incidence. As shown in Figure 4b, the bandwidth of 90% absorption is 0.82 THz at central frequency of 1.68 THz (NBW = 48.8%). The structure supports three graphene plasmon resonances, which cause three absorption peaks (denoted as A, B and C) in the absorption spectrum. In 2018, J. Yang et al. proposed a broadband perfect absorption structure with a flat-top absorption spectrum by coupling monolayer unpatterned graphene with periodical dielectric wires [24]. The schematic image of the proposed structure is shown in Figure 4c, which consists of monolayer graphene sandwiched between periodical dielectric wires and a dielectric substrate on top of a metallic film. Based on the coupling between Mie resonances with graphene plasmon resonances, the bandwidth of Mie resonances and the absorbance of the graphene are improved simultaneously. As a result, the NBW of the demonstrated structure for the absorption above 99% can reach about 60%, as shown in Figure 4d.

In addition, broadband perfect absorption structures based on the periodic structures with graphene-based hyperbolic metamaterials [120], graphene metascreen [121], and graphene plasmonic analogs of electro-magnetically induced transparency [122] have also been studied.

Generally, there is a fundamental trade-off between the operational bandwidth and the attainable absorption, which means it is hard to improve the absorbance and the bandwidth simultaneously. Consequently, most of the broadband perfect absorption structures are based on 90% absorption, and the bandwidth will drop dramatically when trying to obtain higher absorbance. Hence, the broadband absorption structure with higher absorbance needs to be further investigated. The absorption structure in Figure 4c supplies a feasible solution.

### 3.3. Coherent Perfect Absorption

A normal perfect absorption structure just has one port, which means the structure is illuminated from one side by the incident wave. However, perfect absorption can be also achieved in a structure illuminated from both sides, and this two-port absorption is called “coherent perfect absorption” (CPA) [123]. CPA was first demonstrated in a silicon slab [124] and then in a planar metamaterial [125].

**Figure 5:**
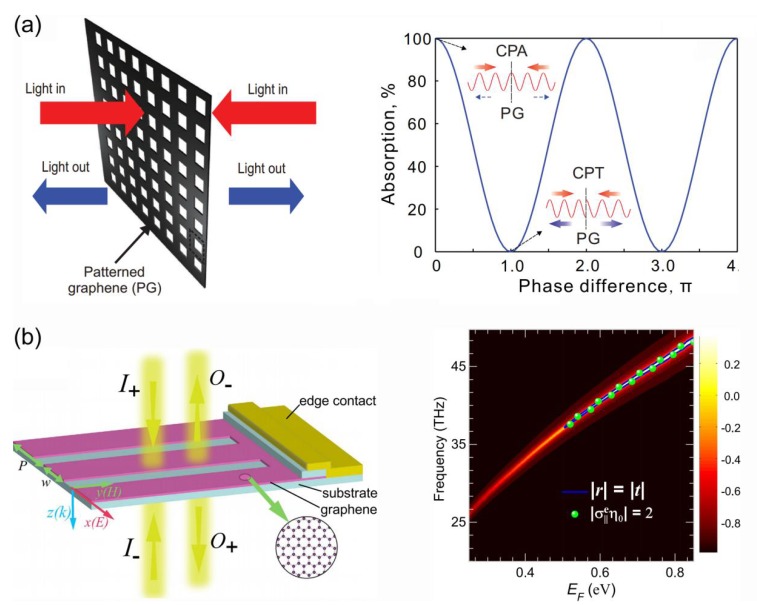
(**a**) Schematic illustration of coherent absorption in a nanostructured graphene, accompanied by the phase modulation of coherent absorption at the resonance wavelength of 8.476 µm [25]. (**b**) Left: schematic of a graphene ribbon meta-surface illustrated by two counter propagating and coherently modulated input beams. Right: The difference (|r|−|t|) of the scattering coefficients for the graphene meta-surface as functions of frequency and Fermi energy *E*_F_, the solid blue line indicates quasi-CPA points where |r|=|t| [126]. Reproduced from references [25,126], with permission from Optical Society of America, 2014; with permission from Nature Publishing Group, 2015.

In 2014, J. Zhang et al. demonstrated that both CPA and coherent perfect transparency (CPT) can be realized in a patterned monolayer graphene in the mid-IR [25]. The schematic illustration of coherent absorption in a patterned graphene is shown in Figure 5a. The absorption of the patterned graphene at wavelength of 8.476 µm can varies continuously from over 99.9% to less than 0.01% when the relative phase of the incident two beams changes from 0 to π, and the modulation contrast is over 40 dB. In 2015, Y. Fan et al. exploited graphene nanoribbons based meta-surface to realize CPA in the mid-IR regime [126]. The schematic of the graphene ribbon meta-surface is shown in Figure 5b, and the CPA in the structure can be electrically tuned in a wide frequency band.

In 2015, H. Xiao et al. utilized split-ring graphene to realize CPA in the THz regime, and the calculated response time of the device was about 36 ps [26]. Meanwhile, graphene-based CPA was demonstrated by using a metal-graphene nanostructure in the mid-IR [127] and CPA in unpatterned graphene was realized in the THz [128,129,130] where doped graphene can be highly conductive and absorptive.

The main advantage of CPA is that optical absorption can be modulated simply by varying the relative phase of the coherent beams. The realization of CPA in graphene may lead to further demonstration of new types of optoelectronic devices such as coherent graphene photodetectors, phase-based sensors or all-optical logical devices.

## 4. Perfect Absorption in the Visible and Near-IR Band

In the visible and near-IR band, the light absorption of suspended monolayer graphene under normal incidence is only 2.3%, and graphene can hardly support plasmon resonances due to the limit of doping level. Thus, the absorption of graphene was normally enhanced by coupling the graphene with metallic or dielectric resonant structures. In the following, we will firstly discuss the perfect absorption structures realized by coupling graphene with metallic nanostructures and with dielectric-mode structures, respectively. We will discuss a chip-integrated graphene-based perfect absorption structure afterwards.

### 4.1. Graphene Coupled with Metallic Nanostructures

Metallic nanostructures can support localized surface plasmon resonances (SPRs) in the visible and near-IR band, and graphene absorption can be effectively enhanced by coupling graphene with metallic nanostructures due to the near-field enhancement. In 2013, S. Song et al. designed a graphene photodetector by coupling graphene with a metamaterial perfect absorber, perfect absorption of the whole structure was realized and the absorption of graphene can be enhanced to above 40% at wavelength around 1500 nm [32]. Similarily, Y. Cai et al. designed an absorption structure in the visible band by coupling graphene with a metal-dielectric-metal perfect absorber, and simulation results show that the peak absorption of monolayer and three-layer graphene are 37.5% and 64.8%, respectively [33]. In order to further increase the absorption in monolayer graphene, B. Zhao et al. proposed a perfect absorption structure by coupling monolayer graphene with a deep silver grating, and peak absorption of graphene can be enhanced to about 80% by exciting the magnetic polaritons or surface plasmon polaritons of the silver grating [34,35].

In 2015, F. Xiong et al. demonstrated graphene-based ultrabroadband absorption by exciting localized plasmons in the metallic structures, and simulation results show that the absorption of monolayer graphene is over 30% in the wavelength range from 780 to 1760 nm [36]. Graphene-based broadband perfect absorption structures were also reported by coupling graphene with gold cylinder arrays [37] or using multiple magnetic dipole resonances of metamaterials [38]. In 2018, Y. Fan et al. designed monolayer-graphene-based broadband and wide-angle perfect absorption structures in the near-IR by utilizing the magnetic coupling effect [39]. Simulation results show that the peak absorption in graphene is 72% and the bandwidth of over 50% absorption in graphene is about 240 nm for a designed structure, and the absorption of the structure is very stable in the incident angle range from 0 to 40 degree.

In addition, dual-band or multiband perfect absorption structures were designed by coupling graphene with metallic nanostructues [40,41,42].

Graphene-based perfect absorption structures designed by coupling graphene with metallic nanostructures have some advantages. Firstly, the *Q* factors of localized SPRs of metallic nanostructures are relatively low, and at the same time, the SPRs can effectively enhance the field intensity in graphene, so the absorption bandwidth of those structures could be broad. Secondly, the frequencies of the SPRs excited by incident waves are normally insensitive to the incident angle, so this kind of perfect absorption structures could have high incident angular tolerance [33,39]. However, this kind of perfect absorption structures also have a big drawback, namely, the high absorption in metals. In particular, the damping constants of nanostructured metallic thin films would be much higher than that of bulk metals, due to the surface scattering and grain boundary effects in thin films [131]. Therefore, the real absorption of metals in the perfect absorption structures could be much higher than the simulation values, which would seriously limit the real absorption of graphene.

In order to reduce the undesirable optical loss, graphene based perfect absorption structures were designed by coupling graphene with dielectric-mode structures, and in the following we will introduce those structures.

### 4.2. Graphene Coupled with Dielectric-Mode Structures

Perfect absorption structures realized by coupling graphene with dielectric-mode structures can minimize the undesired optical loss in metals, and even 100% absorption in graphene can be achieved. In 2012, A. Ferreira et al. and M. Furchi et al. demonstrated that by integrating graphene with two Fabry-Pérot cavities [44] or a Fabry-Pérot microcavity [45], almost 100% absorption in graphene can be achieved theoretically. In 2013, M. A. Vincenti et al. numerically demonstrated that the inclusion of a monolayer graphene in an asymmetric one-dimensional photonic crystal structure can lead to perfect, narrow-band absorption [46]. In 2014, J. R. Piper et al. numerically demonstrated perfect absorption of graphene by utilizing guided resonances of a photonic crystal slab on top of a lossless metallic mirror or a multilayer dielectric mirror [47]. J. R. Piper et al. also designed a perfect absorption structure by coupling a monolayer graphene with a photonic crystal slab without a back mirror, and the perfect absorption in graphene is realized by utilizing the degenerate critical coupling effect [48]. After that, many graphene-based perfect absorption structures in the visible and near-IR band were proposed by utilizing guided resonances in subwavelength periodic structures [49,50,51,52,53,54,55,56,57,58,59,60,61,62,63,64,65,66]. Meanwhile, perfect absorption in graphene can be also achieved by prism coupling [67,68], by using aperiodic multilayer microstructures [69] or by employing coherent illumination [70,71].

Although many perfect absorption structures have been designed, the experimental realization of monolayer-graphene-based perfect absorption in the optical range is still challenging. In 2014, Y. Liu et al. measured peak absorption of 85% at near-IR from monolayer graphene coupled with 2D silicon photonic crystals on top of a back mirror [49]. In 2015, W. Wang et al. demonstrated that the absorption of the monolayer graphene can be enhanced to 77% within the telecommunications band by using Fano-resonant photonic crystals [51].

**Figure 6:**
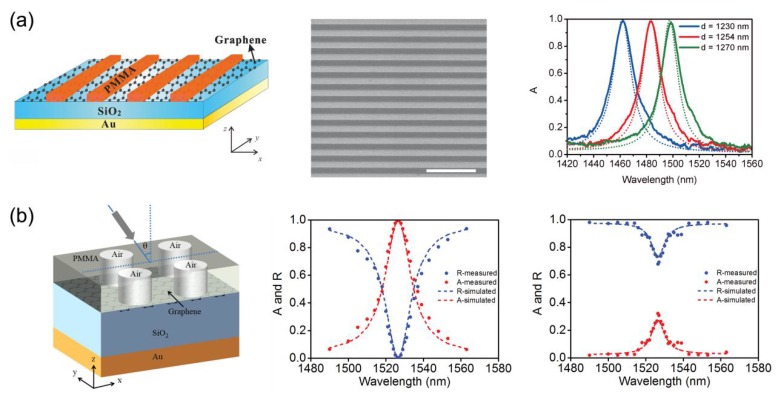
(**a**) Left: schematic image of the perfect absorption structure realized by coupling graphene with 1D PMMA grating. Middle: SEM image of a fabricated structure, the scale bar equals 5 μm. Right: measured (solid line) and simulated (dashed line) absorption spectra of structures with different grating periods under normal incidence of a white light source with TE polarization [64]. (**b**) Left: schematic image of the perfect absorption structure realized by coupling graphene with 2D subwavelength grating. Middle: measured (dot line) and simulated (dashed line) spectra of a fabricated perfect absorption structure. Right: measured (dot line) and simulated (dashed line) spectra of a resonant structure without graphene [65]. Reproduced from references [64,65], with permission from Wiley, 2016; with permission from Optical Society of America, 2017.

In 2016, C.-C. Guo et al. experimentally demonstrated total absorption over 99% in the near-IR for monolayer-graphene-based subwavelength structures, which convincingly confirms the theoretical prediction of graphene-based perfect absorption in the optical range [64]. The schematic image of the perfect absorption structure is shown in Figure 6a. A monolayer graphene is sandwiched between a SiO_2_ layer and a 1D polymethy1-methacrylate (PMMA) grating, and a thick gold layer is coated on the back side of the SiO_2_ layer. A scanning electron microscope (SEM) image of a fabricated structure and measured absorption spectra of absorption structures with different grating periods for TE polarization are also shown in Figure 6a. The measured peak absorption of structures with grating period of 1230, 1254 and 1270 nm are 99.6%, 99.1% and 98.6%, respectively. In 2017, Y. S. Fan et al. experimentally demonstrate a graphene-based perfect absorption structure by coupling monolayer graphene with a 2D subwavelength grating, and the absorption of the structure is polarization-independent at normal incidence [65]. As shown in Figure 6b, peak absorption of 99.4% was measured from a fabricated perfect absorption structure, and the peak absorption of the resonant structure without graphene was around 32%.

### 4.3. Chip-Integrated Graphene-Based Perfect Absorption Structure

**Figure 7:**
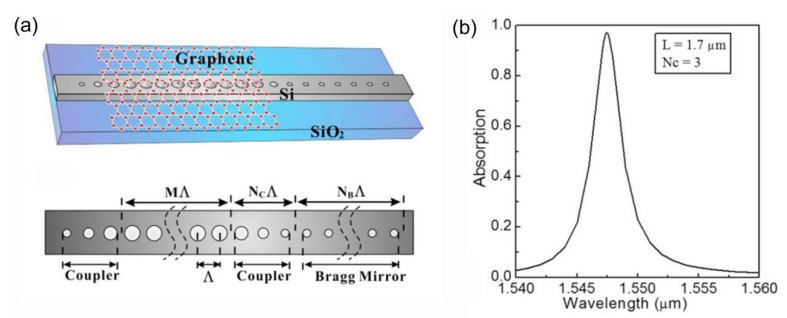
(**a**) Schematic of the waveguide-cavity (covered by graphene)-waveguide (WCW) system. (**b**) The absorption of graphene with a length of 1.7 μm when N_C_ = 3. Reproduced from reference [132], with permission from Optical Society of America, 2015.

The graphene-based perfect absorption structures discussed above are based on free space incidence, which cannot be used for on-chip devices. In 2015, W. Xu et al. proposed a chip-integrated graphene-based near perfect absorption structure through waveguide-cavity (covered by graphene)-waveguide (WCW) system [132]. In order to achieve perfect absorption for a WCW system, both the mode leakage to the output waveguide and the mode radiation loss to the free space should be completely eliminated, and this condition can be hardly satisfied for a practical design. However, nearly perfect absorption can be achieved if the mode leakage rate to the input waveguide is much higher than the mode leakage rate to the output waveguide and the mode radiation loss. The schematic diagram of the designed chip-integrated near perfect absorption structure is shown in Figure 7a. A monolayer graphene is put on top of a silicon-on-insulator (SOI) nanobeam cavity which consists of some uniform air holes in a strip waveguide. Two identical couplers at ends of the cavity are used to control the coupling rate and radiation loss of the cavity, and a Bragg mirror at the output waveguide is used to block the transmission. Figure 7b shows the simulated absorption spectrum of graphene in the structure when the periodicities of the coupler, the cavity and the Bragg mirror are set to be 3, 13 and 10, and the length of the graphene is chosen as 1.7 μm. As shown in Figure 7b, the peak absorption of graphene is 97%.

The theoretical work discussed above may provide an effective way to enhance the performance of chip-integrated graphene photodetectors. However, so far there is no experimental demonstration. Besides, the concept of chip-integrated perfect absorption structure is not limited to graphene and nanobeam cavity, which could also be extended to other WCW systems and other two-dimensional material such as MoS_2_.

## 5. Optoelecronic Applications

Graphene-based perfect absorption structures provide the opportunity to solve the key problem which limits the applications of graphene in practical optoelectronic devices, so they have been applied widely in advanced graphene optoelectronic devices. Here, we briefly summarize some optoelectroinc applications of graphene-based perfect absorption structures.

### 5.1. Photodetectors

Graphene is a promising material for photodetectors since it has ultra high carrier mobility and ultrabroad absorption band. Graphene photodetectors can potentially operate at speeds over 500 GHz [2], and many high-speed graphene photodetectors have been reported [4,5,6,7]. However, the responsivity of demonstrated high-speed graphene photodetectors was relatively low. Although ultrasensitive graphene detectors have been reported by employing the photogating mechanism, the response speed of this kind of detectors was seriously limited [133].

By employing graphene-based perfect absorption structures, we can greatly enhance the responsivity without compromising the operational speed of the graphene photodetectors.

**Figure 8:**
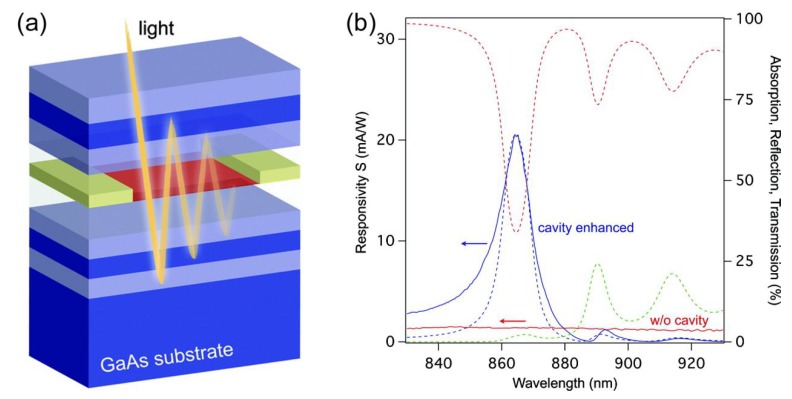
(**a**) Schematic drawing of the graphene microcavity photodetector. Distributed Bragg mirrors form a high finesse optical cavity. (**b**) Spectral response of the bilayer graphene device. The dashed lines show calculation results: reflection R (red), transmission T (green), and absorption A (blue). The solid lines are measurement results: photoresponse for the device with cavity (blue) and photoresponse for the device without cavity (red). Reproduced from reference [45], with permission from American Chemical Society, 2012.

In 2012, A. Ferreira et al. designed a graphene-based photodetector with two Fabry-Pérot cavities, and the peak absorption can reach near 100% [44]. In 2012, M. Furchi et al. demonstrated a graphene photodetector by integrating graphene with a Fabry-Pérot cavity [45]. Near perfect absorption in graphene can be achieved in the simulation for a monolayer graphene microcavity photodetector, and over 60% absorption in graphene was experimentally measured at wavelength of 855 nm. A maximum responsivity of 21 mA/W was achieved for a bilayer graphene microcavity photodetector, which is more than an order of magnitude higher than that of the device without cavity. The schematic image of the demonstrated graphene photodetector is shown in Figure 8a, the device comprises a graphene layer which is sandwiched between two distributed Bragg mirrors. Figure 8b shows the response spectra of a bilayer graphene device. The dashed lines show calculation spectra, and the solid blue line shows measured photoresponse, and the spectral response of a bilayer graphene detector without cavity is also shown in Figure 8b as a solid red line.

### 5.2. Modulators

The optical absorption in graphene can be controlled by tuning the Fermi energy of graphene with electrostatic gating [134]. Based on this mechanism, many electroabsorption modulators based on graphene have been proposed and demonstrated [8,9,10,11,12,13,14]. Graphene modulators have many advantages, and one of the most attracting advantages is the high modulation speed. Theoretical analysis has shown that modulation speeds of graphene modulators can be far beyond 100 GHz [9,10], and modulation speed of 30 GHz has been experimentally realized for graphene modulators at the near-IR [11]. However, due to the low absorption in graphene, the modulation depths of demonstrated graphene modulators are normally poor. Typical modulation depths of spatial and on-chip graphene modulators are 2.1% (~0.1 dB) [12] and 0.1 dB/µm [8], respectively.

**Figure 9:**
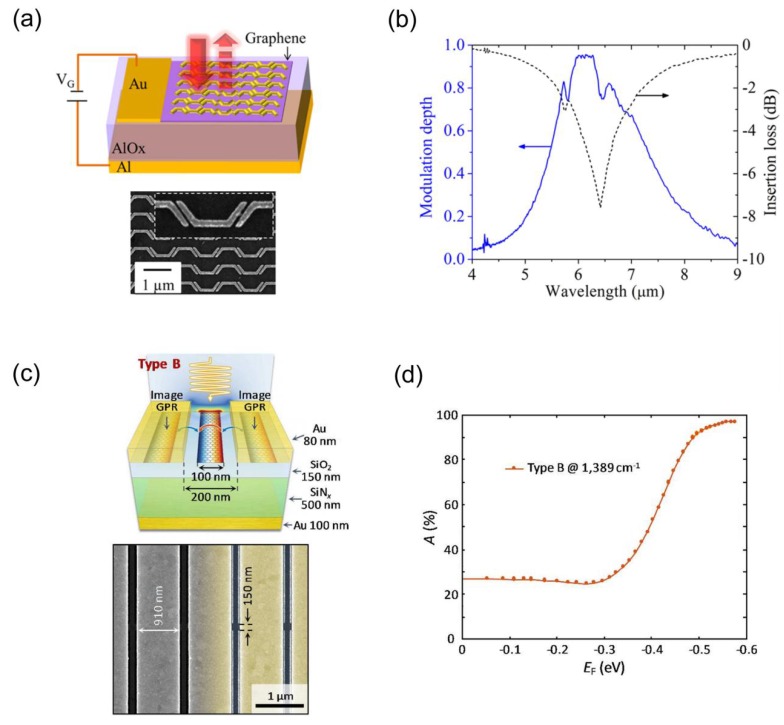
(**a**) Top: schematic of the ultrathin optical modulator based on a tunable metasurface absorber. Bottom: a SEM image of the metasurface on graphene. Inset: a zoomed-in view of a portion of the metasurface. (**b**) The modulation depth achieved experimentally at different wavelength and corresponding insertion loss for the structure shown in (**a**) [72]. (**c**) Top: schematic of a perfect absorption structure incorporating graphene plasmonic ribbons and plasmonic metallic antennas. Bottom: a SEM image of the top structure. (**d**) Absorption of the structure shown in (**c**) as a function of graphene Fermi level (*E*_F_) at the frequency for maximum absorption [73]. Reproduced from references [72,73], with permission from American Chemical Society, 2014; with permission from American Chemical Society, 2018.

Graphene-based perfect absorption structures can greatly enhance the modulation depth of graphene modulators. In 2014, Y. Yao et al. demonstrated graphene spatial modulators at mid-IR by using metasurface perfect absorption structures, and a modulation depth of 95% (13 dB) with modulation speed about 20 GHz was achieved [72]. Figure 9a shows the schematic of a modulator fabricated by Y. Yao et al. and a SEM image of the metasurface on graphene. Figure 9b shows the measured modulation depth and insertion loss of the device as a function of input wavelength. In 2018, S. Kim et al. demonstrated electronically tunable perfect absorption structures by combing graphene plasmonic ribbons with plasmonic metallic antennas [73]. Figure 9c shows the schematic image and a SEM image of a fabricated structure. Figure 9d shows the measured absorption of the fabricated structure as a function of graphene Fermi level. As shown in Figure 9d, the absorption at 1389 cm^−1^ can be tuned from 24.8% to 96.9%, with a modulation depth of 95.9% (13.9 dB) in reflection.

In addition to photodetectors and modulators, tunable optical filters, polarizers and sensors were also demonstrated by utilizing graphene-based perfect absorption structures. The performances of those devices have been greatly enhanced compared with normal graphene-based devices.

## 6. Outlook and Perspectives

The emergence of graphene-based perfect absorption structures could solve the key problem which limits the applications of graphene in practical optoelectronic devices. Until now, a lot of graphene-based perfect absorption structures have been developed in the visible to THz band, and some applications of those structures have been demonstrated by experiments.

Although a lot of works have been done and dramatic progresses have been made in the field of graphene-based perfect absorption structures, the real applications of those structures are still at the early stage. Graphene-based perfect absorption structures have some challenges that need be overcome in the practical device applications. The first challenge is how to simplify the structure which designed to achieve perfect absorption. The fabrication will be a big issue if a designed structure is complicated, and it will be hard to apply the structure in the real devices. In the real applications, unpatterned few-layer structures or planar patterned structures with big feature sizes are preferable, since those structures can be easily fabricated. The second challenge is how to realize near 100% optical absorption in the reality and minimize the undesirable optical loss in the same time. It’s not hard to get perfect absorption in the simulation, but the realization of perfect absorption in the reality is not easy work. For this issue, the designed perfect absorption structure should have relatively high fabrication tolerance and incident angular tolerance. On the other hand, the undesirable optical loss should be minimized. In the visible and near-IR band, the metal attenuation cannot be ignored, so the optical field intensity near the metal should be reduced as much as possible. The third challenge is how to control the wavelength and bandwidth of the perfect absorption. Broad absorption bandwidth is highly desired for photodetection and imaging, and narrow absorption bandwidth with tunable working wavelength is very important for optical filters, spectrally selective photodetectors and sensors. In the mid-IR to THz band, perfect absorption structures based on unpatterned graphene are preferable, in order to avoid the edge effects and simplify the fabrication processes. In the visible and near-IR band, perfect absorption structures realized by combing graphene with dielectric structures are preferable in many cases, since the undesirable optical loss can be minimized. However, the absorption bandwidths of those structures are normally narrow, which are not suitable for broadband applications. The surface mode or surface wave [135] in dielectric structures may provide a solution to broaden the bandwidths of those structures.

In summary, graphene-based perfect absorption structures can greatly enhance the optical absorption in graphene and the light-graphene interaction. We believe their potential is still not fully reached. Besides the applications of photodetectors, modulators, filters and polarizers, graphene-based perfect absorption structures could be also of valuable applications in light emitting devices, photovoltaic devices, nonlinear photonic devices and so on. Graphene has been demonstrated to be a good candidate for light emitters [136,137], however, the radiation efficiency of graphene is very poor. According to Kirchhoff’s reciprocity, the emissivity equals absorptivity, so the perfect absorption structures could greatly enhance the radiation efficiency of graphene. Meanwhile, graphene can be used as the photoactive material in photovoltaic devices, but the low absorption of graphene limits its real application. Broadband graphene-based perfect absorption structures may make graphene photovoltaic devices become a reality. In addition, graphene has very high nonlinear susceptibility, which makes it a good material for ultra compact nonlinear photonic devices. Perfect absorption structures can greatly enhance the efficiency of graphene-based nonlinear photonic devices due to the high optical absorption and high field intensity in graphene.
